# Efficiency of Resistant Starch and Dextrins as Prebiotics: A Review of the Existing Evidence and Clinical Trials

**DOI:** 10.3390/nu13113808

**Published:** 2021-10-26

**Authors:** Michał Włodarczyk, Katarzyna Śliżewska

**Affiliations:** Department of Biotechnology and Food Sciences, Institute of Fermentation Technology and Microbiology, Technical University of Lodz, Wolczanska 171/173, 90-924 Lodz, Poland

**Keywords:** resistant dextrin, resistant starch, prebiotic, human health, gut microbiota, dietary fiber

## Abstract

In well-developed countries, people have started to pay additional attention to preserving healthy dietary habits, as it has become common knowledge that neglecting them may easily lead to severe health impairments, namely obesity, malnutrition, several cardiovascular diseases, type-2 diabetes, cancers, hypertensions, and inflammations. Various types of functional foods were developed that are enriched with vitamins, probiotics, prebiotics, and dietary fibers in order to develop a healthy balanced diet and to improve the general health of consumers. Numerous kinds of fiber are easily found in nature, but they often have a noticeable undesired impact on the sensory features of foods or on the digestive system. This led to development of modified dietary fibers, which have little to no impact on taste of foods they are added to. At the same time, they possess all the benefits similar to those of prebiotics, such as regulating gastrointestinal microbiota composition, increasing satiety, and improving the metabolic parameters of a human. In the following review, the evidence supporting prebiotic properties of modified starches, particularly resistant starches and their derivatives, resistant dextrins, was assessed and deliberated, which allowed drawing an interesting conclusion on the subject.

## 1. Prebiotics

One of the first definitions of prebiotics stems from the mid-1990s, when they were described as “the non-digestible components of the food that facilitate growth and/or activity of a beneficial microorganisms inhabiting the gastrointestinal tract of a host”. In 2007, the World Health Organization (WHO) decided to update said definition by describing prebiotics as “non-viable food components that have a health benefit on the host by modulation of the microbiota” [1]. Today, compounds must meet number of criteria to become a certified prebiotic. Firstly, they must be highly resistant or completely immune to the acids and enzymes of the gastrointestinal tract. Secondly, it is crucial that prebiotics are exclusively used as a fermentation substrate by a chosen group of beneficial gut microorganisms. Furthermore, it is required that a prebiotic promotes the growth and increase the counts of the favorable bacteria while ideally decreasing the amount of less-desired ones. The last condition, although equally important, is that prebiotic compounds must be viable after food processing such as thermal, chemical, or physical treatments [2].

Prebiotics are usually plant-derived oligosaccharides, essentially fructans, galactans, or similar compounds [3,4,5]. In the group of fructans, fructooligosaccharides (FOS) and inulins are found, while galactans include galactooligosaccharides (GOS). Starch derivatives such as resistant dextrins, or compounds such as xylooligosaccharides, pectins, and beta-glucans, similarly comply with the prebiotics’ recognition criteria [6,7]. Many prebiotics can be easily found in nature. The list is long, but some of the most common sources of prebiotics include foods such as cereals, oats, wheat, barley, various berries, onions, garlic, bananas, tomatoes, legumes, and honey [8]. Moreover, prebiotics are often used as food additives to increase products’ nutritional value [9].

**Figure 1:**
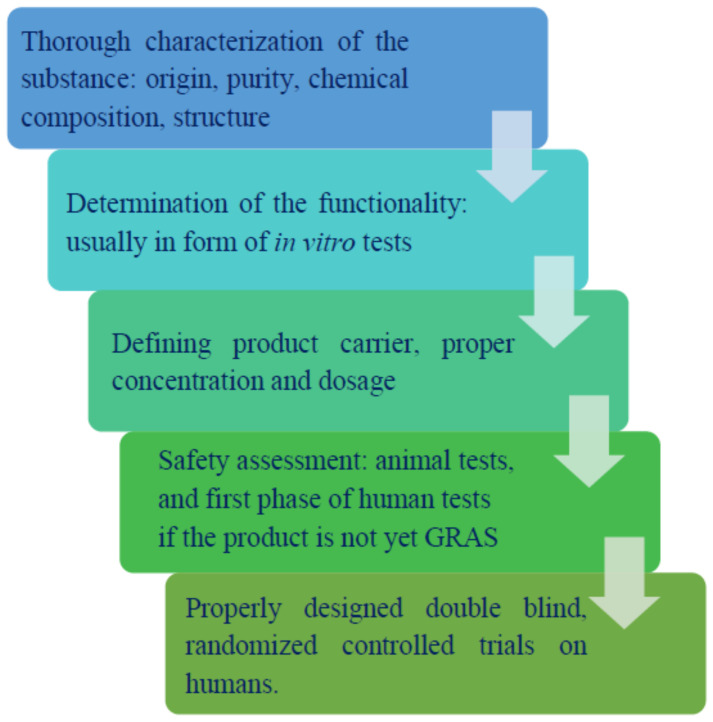
Steps of prebiotic recognition.

Nonetheless, it should be noted that not all fiber sources are considered prebiotics. The European Food Safety Authority (EFSA) strongly differentiates the terms dietary fiber and prebiotic by specifying that distinctive products cannot be classified as prebiotics, but only as a dietary fiber, if “a cause-and-effect relationship has not been established between the consumption of the food constituents, which are the subject of the health claims and a beneficial physiological effect related to increasing numbers of gastrointestinal microbiota” [10]. Moreover, there are certain measures involved in the process of prebiotic recognition that require time and specifically designed clinical trials proving the beneficial effects of a selected substance (Figure 1). Thus, even if a given substance exhibits seemingly prebiotic properties, it may take years of testing to prove it.

**Figure 2:**
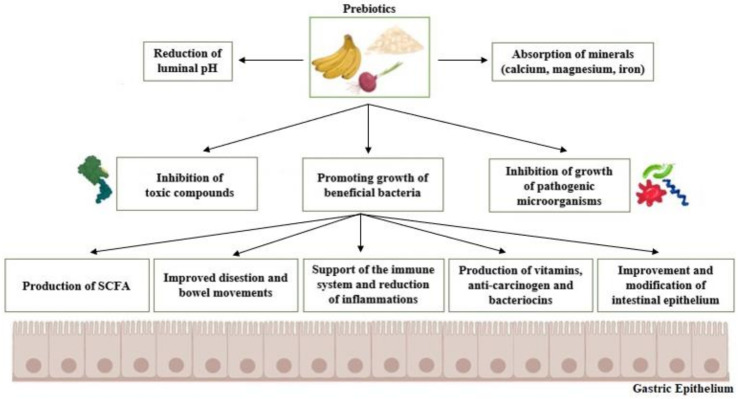
Basic mechanisms of action and benefits of prebiotics. SCFA: Short chain fatty acids.

Prebiotics per se do not possess many outstanding health-promoting properties; instead, they are an important substrate of fermentation for the beneficial species of microorganisms present in the host’s gastrointestinal tract and thus induce their growth (Figure 2). Then, the desired positive effects take place. Those include the secretion of useful and beneficial metabolites, production of the short-chain fatty acids (SCFA), assistance in ion absorption processes (i.e., cations of magnesium, calcium, and iron), and the improvement of the performance of the host’s immune system performance (i.e., by increasing immunoglobulin concentrations and regulating cytokine production) [4].

Valuable microbial genera present in the intestines, namely *Bifodobacteria* and *Lactobacillus* spp., are able to utilize saccharides as a carbon source by means of fermentation. Desired byproducts of their metabolism include SCFAs, which are associated with various beneficial effects noticeable in the host’s organism, i.e., control of appetite by decreasing the concentrations of ghrelin [11]. Moreover, increased levels of SCFAs lead to reduction of the body fat accumulation by altering the energy expenditure and intake, elevating the oxygen consumption rates, and increasing the thermogenesis and oxidation of fat [12,13].

Furthermore, a recent study by T. Cai et al. (2021) investigated the effects of *Lacticaseibacillus casei* DG^®^ on the treatment of chronic bacterial prostatitis with exceptionally promising results [14]. Over 70% of tested patients (61 subjects) confirmed the reduced symptoms and recurrences of the disease. Moreover, it was proven that *L. casei* DG^®^ significantly decreases the usage of antibiotics during the treatment, which is likewise economically positive, given that it potentially leads to lower costs of therapies.

Several studies on prebiotics highlighted their promising potential for the absorption of microelements such as calcium, magnesium, zinc, copper, iron, and phosphorus. This effect is associated with an increased count of gut microbes (*Lactobaliccus* and *Bifidobacterium* spp.) and the production of SCFAs, which modulate the intestinal pH, thus improving the solubility of minerals, making them easier to assimilate [15]. It was additionally found that SCFAs can induce expression of proteins able to bind calcium [16]. It is also suspected that fiber compounds possess the ability to increase the water retention capacity of epithelial cells, which enlarges their area and enhances the ability to absorb minerals [17].

Prebiotics can furthermore aid the host’s immune system in several ways: directly through immune cell stimulation (by β-glucans) or indirectly through the production of SCFA, which contributes to an increased activity of immune cells (T-helper cells, killer cells, and macrophages) [18,19,20]. Another indirect benefit is associated with competition between potentially pathogenic strains invading the gastrointestinal tract and beneficial species of bacteria (i.e., probiotics) [21]. They bind to the epithelial cells and the mucus, forming a barrier for pathogens (competitive exclusion), and secrete metabolites, which can suppress the activity of invading microorganisms. Moreover, due to their close interaction with the intestinal cells, the immune response can be stimulated [22]. There are several studies proving the positive correlation between the treatment and prevention of colorectal cancer and the gut microbiota, mainly *Lactobacillus* and *Bifidobacterium* spp. Increased levels of abovementioned species can boost the immune system and even inhibit the formation of cancer cells [23,24]. Properly regulated intestinal microflora may be considered as a factor in processes of inactivation and the removal of toxic compounds, help to reduce inflammations, and significantly enhance the immune responses [25]. Prebiotics were also proven to be a viable support in the treatment of the inflammatory bowel disease (IBD) by inducing the growth of beneficial bacteria, thus elevating the concentrations of SCFA, which boost the host’s immunological responses [26].

An important role of prebiotics is the inhibition of toxins in the intestines. Bacteria grown during the prebiotic treatment were shown to protect the colon lumen by adhering to its surface and reducing the effect of toxic carcinogenic compounds such as N-methyl-N′-nitro-N-nitrosoguanidin or 1,2-dimethylhydrazine. The exact effect of the latter is not fully described yet, but it is suspected that lactic acid bacteria (LAB) may secrete metabolites that inactivate or scavenge some of the carcinogenic intermediates [27,28]. It is furthermore known that the activity of some fecal enzymes (nitroreductases, β-glucuronidase, and β-glucosidase) is directly associated with the production of mutagenic compounds (i.e., aglycons production due to β-glucuronidase activity). Studies show that strains of LAB possess the ability to reduce the activity of these harmful enzymes [28,29].

## 2. Dietary Fibers

The definition of dietary fiber (DF) and its effects on health have been widely discussed topics over the years. In 2008, the most recent and uniform definition was issued after the Codex Alimentarius [30,31]. Accordingly, DF is comprised of carbohydrate polymers with at least 10 monomeric units (MU), which can neither be digested nor absorbed in the human’s intestines. However, it is stated that national authorities can decide to include carbohydrates with lower MU number ranging between 3 and 9, which is the case in the EU as well as in several countries e.g., Australia, Canada, Japan, and the USA [30,32].

When properly extracted (chemically, physically, and/or obtained enzymatically or synthetically) and purified, the generally recognized scientific evidence of health benefits must be proved to define a polymer as DF. Respective definitions often include other ‘associated substances’ that are not carbohydrates, e.g., lignin [30].

These views are evident in the worldwide dietary recommendations, which strongly emphasize the consumption of DFs naturally occurring in food, whereas added and nondigested carbohydrate polymers are much less recommended, due to the limited evidence of health benefits of these specific types of fiber.

Regarding the benefits for health, it is indicated that DF must generally present one or more of the following properties:

➢ reduce the stool transit time, while increasing its bulk [33];
➢ be used as a carbon source by colonic microbiota (as fermentation substrate) [34];
➢ reduce the levels of cholesterol (either total or LDL cholesterol) [35]; and
➢ support glucose and insulin metabolism (reduce the levels of blood glucose and insulin) [36].

These, among other related criteria, were presented in the EU Directive 2008/100/EC and were recently used for the evaluation of the health-promoting properties of a large number of fibers by the FDA [32] and Health Canada’s Food Directorate [37]. According to the abovementioned directive, it was concluded that most of the currently available compounds commercially called ‘dietary fibers’ can be officially defined as dietary fibers. The list of approved fibers include:

➢ non-starch polysaccharides including cellulose, hemicelluloses, mannans, pectins, and other hydrocolloids (i.e., b-glucans, gums, and mucilages), inulin, and fructans;
➢ resistant oligosaccharides including fructo-oligosaccharides (FOS), galactooligosaccharides (GOS);
➢ resistant starch and dextrins [30].

Purportedly, the number of approved fibers will rise over time, as more studies will provide sufficient evidence for the health benefits of dietary fiber’s candidate compounds. Considering the role of the colonic fermentation of fiber as the source of its health-promoting properties, some differences exist among countries. According to the FDA, the applicable fermentation-related criterium is “increased mineral absorption”, which is associated with the increased solubility and bioavailability through the process of production of SCFA. Moreover, the “reduction of energy intake” strongly correlated with the colonic fermentation of fibers is likewise considered a valid criterium [32].

In the EU, the process of evaluation and recognition of dietary fiber is usually conducted using analytical criteria through the commonly acknowledged AOAC 2009.01 standard method or other similar methods (Table 1).

**Figure 3:**
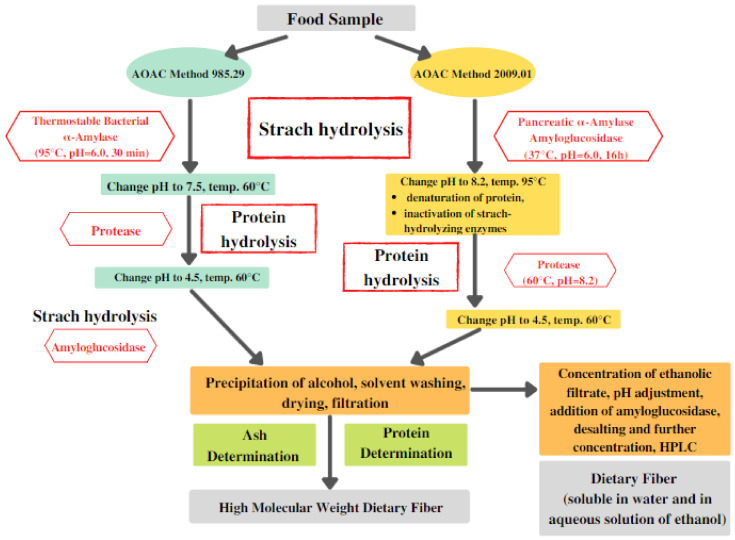
Main steps involved in the determination of dietary fiber. AOAC: Association of Official Analytical Collaboration; HPLC: High-performance liquid chromatography.

In most of these methods, thermostable bacterial α-amylase, protease, and amyloglucosidase are used. However, due to the high temperature of amylase incubation (95 °C), there are notable losses in resistant starch since it undergoes hydrolysis in such conditions. On the other hand, AOAC methods 2009.01 and 2011.25 both employ incubation with pancreatic α-amylase under specific conditions that are comparable to physiological ones (37 °C, pH = 6.0); thus, they offer a much more accurate estimation of resistant starch [38]. A general scheme presenting two main methods of dietary fiber determination is presented in Figure 3.

For these methods (Figure 3), it is recommended to use the purest enzymes possible to avoid any type of undesired contamination of the samples. It is also important to verify if the commercial amyloglucosidase preparation is contaminated with cellulases (e.g., β-glucanase), as this is often the case [38,39]. If overlooked, such contamination can lead to depolymerization, which will result in significant underestimation of dietary fiber in food products. Another factor of high importance is the activity of the employed enzymes [39]. Before conducting tests, it is recommended to assess the activity of commercial enzyme given that it can be lower than the official one required for the dietary fiber determination. For example, in the case of low-activity protease, the protein hydrolysis can be partial, which will result in a high content of residues affecting the accuracy of the test [38].

It was proven that several types of dietary fibers are completely fermentable, or at least to a certain degree [30]. The main distinction is that several dietary fibers are fermented rather rapidly by the intestinal microorganisms, while some undergo fermentation at a much slower pace, or in some cases, to a lower degree [40,41].

Dietary fibers have varying characteristics; therefore, various classifications are used to appropriately characterize them. They include origin, chemical composition, and physicochemical properties with subcategorization including the degree of polymerization. Notably, these properties can have a significant impact on the fermentation processes [42].

According to their origin, plant-originated fibers can be divided into the ones derived from fruit and vegetables, cereals and grains, nuts, and legumes. Nevertheless, it must be noted that the different types of plant origin will result in significantly distinct chemical compositions of dietary fibers and different physicochemical properties [43,44]. In the case of bananas, for example, the main fibers are resistant starch and inulin-type fructans, whereas apples are considered a viable source of pectins [45,46]. Even though dietary fibers are easily found in nature and many functional foods (beverages, bakery products, and meats), there are also multiple supplements offering them in an accessible form of powder or tablets (Table 2) [47]. However, it must be noted that many of those preparations are not comprehensibly tested and should be used carefully. Considering these facts, it is crucial to have a balanced and diverse diet incorporating several types of plant-based foods that grant various types of dietary fibers, promoting the growth of particular kinds of intestinal microorganisms [48,49].

The main physicochemical properties of dietary fibers consist of fermentability, viscosity, and solubility. All of them can alter both fermentation processes and the overall effects of fiber product consumption [50].

Insoluble fibers are usually less used for microbial fermentation (e.g., cellulose), but their presence in the gastrointestinal tract elevates the food transit rate and prevents constipation [34].

Another non-fermentable fiber is psyllium, which is known for its increased solubility and high viscosity. Combining these properties proved to be useful in the treatment of high blood cholesterol and in improving the glycemic control of patients [51,52,53].

β-glucans and pectins are fibers that are easily fermentable by gut microbiota while possessing high solubility and viscosity, similarly to psyllium [50]. These fibers occur naturally in whole grains and fruits: β-glucan occurs mainly in barley and oats, whereas pectin occurs in lemons and apples [43].

Fibers with properties such as low viscosity and high solubility can be rapidly used by the gut microbiota for fermentation purposes: this includes resistant starch and dextrins, inulin, polydextrose, and absoluble type of corn fiber [54]. Inulin-type fructans are commonly found in agave, asparagus, onions, artichokes, chicory root, bananas, garlic, leeks, and wheat [55].

Although properties including degree of polymerization or the origin of inulin-type fructans have proven to influence the fermentation processes in patients, there is still not enough evidence confirming their physiological or metabolic benefits [56]. On the other hand, studies on rodents showed that incorporating inulin-type fibers into the diet can reduce the total body weight, fat tissue, and concentrations of blood cholesterol and glucose and stimulate the immune system [57,58].

The solubility of carbohydrates impacts the place in the gastrointestinal tract where the microbial fermentation occurs. Fibers such as FOS or pectins that possess relatively high solubility can be used by the bacteria residing in the gastrointestinal tract (i.e., ascending colon). On the other hand, less-soluble fibers such as cellulose are often metabolized partially in the distal colon because of their significantly slower transit time and the higher densities of bacteria present there [59,60].

## 3. Short-Chain Fatty Acids

A number of studies highlighted that many positive effects attributed to high-fiber diets are actually directly linked to the short-chain fatty acids, which are produced by the gastrointestinal microbiota via the fermentation of ingested oligosaccharides [61,62,63,64].

It was proven that there is a direct correlation between the consumption of prebiotics and dietary fibers with elevated SCFAs production in the intestines and high peripheral circulating SCFAs [65]. Various clinical studies on both human subjects and animals have confirmed that the consumption of prebiotics and dietary fibers has multiple positive effects on the hosts organism, e.g., can improve glucose response after a meal or significantly increase the diversity and counts of butyrate-producing bacteria in the gastrointestinal tract [66], enhance metabolism of glucose, elevate SCFAs concentrations in the serum [67], increase glucose-stimulated insulin secretion (GSIS), and decrease the levels of intestinal endotoxin and pro-inflammatory cytokines [68].

**Figure 4:**
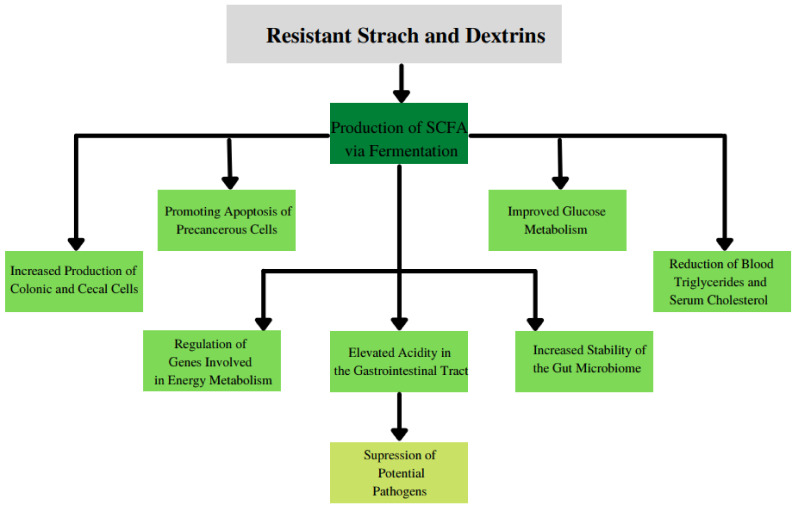
The main benefits of consumption of resistant starch and dextrins.

Some of the main benefits of prebiotics are presented in Figure 4.

The idea that the SCFAs and energy homeostasis are correlated has been investigated thoroughly over the past years. Recent studies have provided an abundance of evidence for the multi-level network in which SCFAs can utilize their beneficial actions such as improving lipid, glucose, and cholesterol metabolism [69,70]. The importance of these correlations is much more evident when the balance of intestinal microbiota is disturbed, which results in the development of diabetes, inflammations, insulin resistance, or obesity [70,71].

### 3.1. Effects of SCFA Absorption in the Human Colon

In the human organism, three main SCFAs are produced by fermenting gut microbes, namely acetate, propionate, and butyrate, which constitute about 95% of SCFA content in the gastrointestinal tract [72,73]. The SCFAs are mainly produced in the colon, since the highest concentration of oligosaccharides, which are the main substrate for fermentation, exists there in the form of undigested foods such as dietary fibers, prebiotic substances, etc. Interestingly, although the main place of SCFA production is the proximal colon, the infusion of acetate to the distal colon of the obese patient was able to increase fasting fat oxidation, with a significant rise in the concentration of plasma peptide YY and post-meal insulin, which suggests that the SCFAs concentrations in the distal colon may possibly play a vital role in enabling their proper functions [74]. After SCFAs are produced in the gastrointestinal tract by the bacteria, they undergo absorption by epithelial cells or are used as an energy source by the liver (mainly butyrate) [70]. However, not all primary SCFAs are absorbed equally fast. Butyrate and propionate undergo efficient absorption by epithelial cells, whereas acetate is assimilated more slowly [75]. It was estimated that about 5% to 10% of SCFAs formed in the intestines are excreted with feces [76]. In the literature, several mechanisms for the process of colonic absorption of SCFAs have been proposed, which are non-ionic diffusion, exchange with bicarbonate [77,78], and co-transportation with cations by the hydrogen-coupled monocarboxylate transporters (MCT1, MCT2, and MCT4) [79] and also by the sodium-coupled monocarboxylate transporter 1 (SMCT1) [80]. Via these mechanisms, SCFAs can influence the pH of the lumen and the volume of the epithelial cells (butyrate and propionate). Moreover, the absorption of Na^+^ and Mg^2+^ cations is increased by butyrate and propionate, respectively [81]. Acetate and propionate also possess the capacity for appetite regulation (by stimulating PYY and GLP-1 secretion), which is proven to have a positive effect on body composition [75]. Moreover, a study by Binder (2010) has shown that decreased SCFA production (for example, by antibiotic-induced reduction of the colonic microbiota) can result in diarrhea, while daily administration of prebiotic supplement or dietary fiber can be used as the effective therapy to support the treatment of acute diarrhea through stimulation of the SCFAs production in the colon and by enhancing the absorption of sodium [82]. Additionally, butyrate oxidation can contribute over 60% of the oxygen consumption in humans’ both descending and ascending colons [83].

### 3.2. SCFA Receptors

In the early 21st century, the SCFAs sensing receptors (GPR41 and GPR43) were discovered and described [84], SCFAs were considered to be the signaling molecules participating in several cellular processes such as ionic transport and activation of transcription factors [81]. GPR41 and GPR43 receptors have been established as necessary for mediating various SCFA effects [85]. Several studies investigated the positive effects of SCFA on the regulation of appetite and energy homeostasis [75,86]. SCFAs may affect the host’s metabolism via mechanisms that directly involve GPCRs or are independent of them [87]. For example, the study of Park et al. (2015) showed that the effect of SCFAs on effector T cells is independent of GPR41 and GPR43 receptors. Furthermore, the same study discovered that the SCFAs are able to inhibit the histone deacetylase activity without direct use of GPR41 and GPR43 receptors [88]. Based on those findings, it would be advisable to further investigate the dependencies between the SCFAs and their receptors and the host’s metabolism and immune system.

### 3.3. Influence of SCFA on Gut-Brain Axis

It was discovered that free SCFAs are able to use monocarboxylate transporters to transmit the current state of the intestines to the brain similarly to signaling molecules [89].

Research performed by Frost et al. (2014) indicated that SCFAs produced by gut microbiota can act as appetite-regulating agents [90]. The mechanism of this process suggests the involvement of neuronal activation induced by the acetate in the arcuate nucleus in the hypothalamus region, where significantly decreased hypothalamic AMPK activity was noticed, together with increased activity of acetyl-CoA carboxylase [90].

The intestine is capable of the secretion of hormones (formed in the gut) that can convey information about the nutritional and energy status of the gut directly to the brain [91]. The discovery of regulatory effects of SCFAs on several gut-derived hormones established them to be a crucial factor in the food intake regulation in humans by appetite modulation [92]. Several studies (in vitro and in vivo) demonstrated that the secretion of the GLP-1 and PYY can be induced by the SCFAs [13,92]. Moreover, elevated concentrations of GLP-1 and peptide YY after ingestion of SCFAs were able to increase the effects of SCFAs on secretion of the gut-derived hormones [13,93]. A number of clinical human trials further demonstrated the effect of SCFAs produced by intestinal microbes on secretion of the gut hormones [93,94,95]. Additionally, healthy subjects who consumed an additional amount of inulin (and thus had increased concentrations of SCFAs) exhibited significantly elevated GLP-1 concentration in the plasma 0.5 h after the administration and notably decreased ghrelin concentration after a test meal that occurred just a few hours after the first administration [65]. 

Interestingly, a direct administration of propionate to the human colon led to similar results (decreased ghrelin and elevated PYY), which further confirmed the role of SCFA in the regulation of appetite hormones [96].

### 3.4. Functions of SCFA in the Liver

The liver is a vital organ, especially for the absorption of propionate and butyrate [76,97]. The lack of balance between the formation and breakdown of lipid molecules, as well as glucose and cholesterol metabolism, can easily result in negative alteration of liver energy metabolism [98]. In the liver, the SCFAs’ metabolism may have a direct effect on energy status as they are turned into sources of energy. A simple example is propionate, which may be easily transformed to glucose via gluconeogenesis in the liver by engaging in the tricarboxylic acid cycle [99]. It was further revealed that the dietary SCFAs can activate a “switch” from hepatic lipogenesis to hepatic beta-oxidation, thus reducing hepatic steatosis, elevating energy expenditure, and serving as protective mechanism against high fat diet [100]. It is moreover suggested that the activation of the UCP2-AMPK-ACC pathway is necessary for these SCFA-mediated beneficial effects on hepatic metabolism [100].

SCFAs can also influence glucose metabolism in the liver [101]. In an animal study, where rats were receiving an acetate-rich diet (0.2% *w*/*w*), a significant increase in the glycogen, citrate, and lower xylulose-5-phosphate concentrations in the liver was observed, which suggests that the inactivation of synthesis of fructose-2,6-bisphosphate induces processes such as acetate-activated gluconeogenesis and acetate-inactivated glycolysis [102].

SCFAs were also able to significantly reduce the synthesis of total cholesterol in the liver [103]. Supplementation with SCFAs for 6 weeks resulted in a significant decline in liver total cholesterol synthesis and in concentrations of plasma cholesterol when compared to groups without prebiotic diets [104]. It was also demonstrated that by using liposome encapsulated acetate (LITA) to externally deliver acetate, it was possible to reduce the accumulation of lipids, lower lipogenesis, and elevate mitochondrial functions in the liver of tested mice. Therefore, it can be suspected that anti-lipogenic properties of SCFAs in the liver might be self-sufficient and work independently of other surrounding processes [105].

Nevertheless, studies mentioned in this section were conducted on animals (mostly rodents), so they only give a view of how SCFAs could potentially have similar metabolic effects on humans. Studies on humans in this area are limited, but as the potential applications are visible, this topic should be investigated thoroughly in the upcoming years [106].

#### 3.4.1. Resistant Dextrins in Clinical Trials

Over the last decade, the topic of new fiber preparations has received great attention. As a result, many prebiotic candidate products were developed by physical and chemical modification of starch, such as type 4 resistant starch (RS4) or resistant dextrins (RDs).

By definition, the RDs are short-chain glucose polymers that lack a sweet taste but exhibit increased resistance to the enzymatic hydrolysis by the digestive enzymes of human [107].

The basic methods of RD production include starch dextrinization, which replaces default 1,4- and 1,6- glycosidic bonds in starch with 1,2- and 1,3- glycosidic bonds [107]. This phenomenon occurs when starch is exposed to high temperature and specific acidic catalysts, which cause chemical reactions such as trans-glycosylation, depolymerization, and repolymerization [108]. High temperatures (over 100 °C) cause random hydrolysis of 1,4- and 1,6- glycosidic bonds in starch, which causes the formation of hemiacetal or aldehyde groups, which then are able to react randomly with active -OH groups of glucose to finally form 1,2- and 1,3- glycosidic bonds [107]. After these modifications, the obtained dextrins with new chemical bonds become significantly more resistant to enzymatic digestion in the human gastrointestinal tract simply by reducing the amount of bonds that can be targeted by digestive enzymes [109].

Nutriose is supposedly one of the most popular commercially available resistant dextrins concluding from the number of studies using it. It is a soluble fiber of low viscosity made from wheat, maize, or pea starch through a highly regulated dextrinization process accompanied by chromatographic fractionation [110]. It exhibits high resistance to the activity of digestive enzymes present in the small intestine, while being fermented to a great extent in the large intestine. About 75% is used for fermentation purposes in the colon, whereas only about 15% of Nutriose is digested and absorbed in the small intestine, and 10% is removed with feces. Such properties confirm it has as high of a potential as prebiotic fiber, as it can primarily be used by the gut bacteria for fermentation [49]. Moreover, Nutriose constitutes up to about 20–25% of a commercial food product’s composition without causing undesirable effects such as bloating and general discomfort [111]. Nutriose has several proven beneficial effects such as elevating the production of SCFA, increasing concentrations of α-glucosidase in feces, increasing short term satiety, and inducing growth of health-beneficial bacteria, i.e., *Bacteroides* while decreasing *Clostridium* spp. [49,112,113].

Nutriose has a variety of applications in the food and pharmaceutical industry, as a valid ingredient of drinks and supplements enriched with fiber [114], granulation binder [115], and a component of dietetic foods lower in sugar and calories [116].

Another commercially available dextrin preparation is Fibersol-2, which is a resistant maltodextrin with prebiotic properties produced from corn-originated starch. Due to specific treatment, it becomes highly resistant to digestion in the small intestine, contrary to the typical maltodextrin [117].

The main production procedure of Fibersol-2 includes pyrolysis and enzymatic treatment of corn starch detailed in US Patent Nos. 5620873 and 5358729. In the first step, the hydrochloric acid is used to perform a transglucosidation reaction in corn starch. Then, the obtained mixture is hydrolyzed by an amylase, purified, and analyzed in order to confirm the proper quality. Finally, it is spray dried to obtain the final product.

The abovementioned steps lead to conversion of part of the original α-1,4 glycosidic bonds to random α-1,2- or 1,3- bonds. Thus, the final product contains not only α-1,4 and 1,6 glycosidic bonds typical for starch but also α-1,2- or 1,3- bonds [118].

According to the manufacturer, Fibersol-2 is well-soluble in water (even 70% *w*/*w* at 20 °C); it does not produce any kind of clods, only clear and transparent water-like solutions; therefore, it is a perfect additive to several types of healthy drinks. Moreover, Fibersol-2 has no flavor or odor and minimal-to-no impact on the sweetness of products. It is stable in a variety of food processing conditions such as high temperatures or acidity and therefore can be used in products such as sauces, milk drinks, yoghurts, sports drinks, puddings, juices, and other similar products. Other features of Fibersol-2 include low hygroscopicity, very low viscosity, and high freezing–thawing stability [117]. While fermented, Fibersol-2 produces less acidic compounds and significantly smaller volumes of gas compared to other conventional dietary fibers [119].

Studies showed that Fibersol-2 has several health-promoting properties such as the reduction of blood glucose and postprandial insulin levels [120] and the decrease of blood triglycerides and serum cholesterol [121], maintaining proper function and health of the colon by moisturizing and speeding up transit of stools (which potentially limits the occurrence of colon diseases and development of cancers) [122]. Moreover, Fibersol-2 was able to promote the growth of several probiotic species while indirectly acting as a suppressor of growth of potentially pathogenic or unwanted microorganisms [123,124].

In the presented clinical trials (Table 3), the supplementation of resistant dextrin had multiple positive effects on various health markers, from the improvement of body weight and BMI of subjects to significant metabolic and immunological benefits. Ingestion of resistant dextrin also promoted the growth of important gastrointestinal microbiota, involved in the production of SCFA, e.g., butyrate and propionate. Therefore, it could lead to the activation of GPCRs and free fatty acid receptors, which results in the elevated secretion of PYY, GLP-1, and gastric polypeptides with inhibitory properties. Butyrate alone promotes the expression of peroxisome proliferator-activated receptor gamma, which improves fatty acid oxidation in the muscle tissue, leading to decreased insulin resistance. Other benefits of the resistant dextrin according to the clinical trials are increased GLP-2 hormone concentrations, reduced endotoxin levels, and inflammations.

Unfortunately, the number of tested resistant dextrins is very limited, and the majority of the clinical studies used Nutriose or Fibersol-2, as they are well-known and available resistant dextrins. Nevertheless, it is greatly advised to conduct further research on different kinds of resistant dextrins to further prove their prebiotic potential. Additionally, there might be differences in the effects of RDs dependent on the gender, but currently the studies targeting said issue are very limited. Therefore, until proper investigation is conducted, no certain conclusions can be drawn.

#### 3.4.2. Overview of Resistant Starch

Starch is a common carbohydrate found in several food products, such as cereals and potato-like plants, and in the organs of some tropical plants. As a result of digestion, 1 g of starch provides the organism with energy equivalent to approximately 4 kcal (16.7 kJ) [145]. However, it has been observed that certain parts of starch are incompletely digested after consumption and were able to remain intact or undergo partial hydrolysis in order to pass through the small to the large intestine [146]. For this special kind of starch, the term “resistant starch” (RS) has been developed. By definition, “resistant starch is the sum of starch and products of its degradation not absorbed in the small intestine of a healthy human” [107]. Resistant starch can likewise be described as the difference between the amount of starch exposed to the action of amylolytic enzymes and the amount of starch that was converted to glucose during its hydrolysis. Accordingly different types of starch can be included in the equation [147].

RS = TS − (RDS + SDS)
RS1 = TS − (RDS + SDS) − RS2 − RS3
RS2 = TS − (RDS + SDS) − RS1 − RS3
RS3 = TS − (RDS + SDS) − RS1 − RS2

where:

RS—resistant starch

RS1, RS2, RS3—resistant starch type 1, 2, 3, respectively

TS—total starch

RDS—quickly digestible starch

SDS—slowly digestible starch

Resistant starch has been defined in five forms [6]. RS1 is physically inaccessible starch; RS2 is the starch of raw (non-gelatinized) granules of certain plant species; RS3 is starch that underwent a retrogradation process; RS4 is chemically or physically modified starch, RS5—starch complexes, such as starch–fatty acid or starch–monoglyceride, have emerged as a separate subclass [148]. Some basic information about various starch types were presented in Table 4.

#### 3.4.3. Resistant Starch Type I

Type 1 resistant starch (RS1) refers to starch molecules inside plant cells with undamaged cell walls. This starch cannot be reached by amylolytic enzymes, since the enzymes of the gastrointestinal tract are unable to degrade cellulose, hemicelluloses, and lignins, which are common components of plant cell walls. That is why RS1 can pass the small intestine without being digested [6].

#### 3.4.4. Resistant Starch Type II

Resistant starch of the second type (RS2) constitutes granules of raw starch of certain plant species, e.g., potatoes or green bananas. Its resistance to the activity of digestive enzymes has not been completely described yet.

It appears, however, that the reason for different susceptibility of potato starch to amylolytic enzymes to starches of other cereals can be caused by significantly lower ability of the enzymes to attach to the potato starch granules compared to that of cereal starches [6,149].

Another feature of potato starch is a relatively high amount of amylopectin together with rather high degree of crystallization. Since the amylolytic enzymes target the amorphous regions first, their activity might become significantly impaired if they encounter the crystalline structure of starch granules. Nevertheless, the reason underlying the resistance of starch to activity of amylases is not only linked to the degree of starch crystallization. For example, cereal starches, which are characterized by a type A crystallization of relatively high degree, are less resistant to the enzymatic activity than potato starch of type B with even a two-times-lower crystallization degree. Interestingly, the starches of legumes, which possess a mixed crystalline pattern (type C, which is a mixture of types A and B), likewise present a high resistance to enzymatic hydrolysis [149].

The resistance of starch granules can be increased by annealing, which happens when the starch is kept in water with a temperature lower than that of gelatinization. Generally, when the water temperature around the starch granules reaches 20 °C, they increase their volume (by approximately 30%). When starch is exposed to an increased temperature, water starts to penetrate the interior of the granule, thus enlarging it. As a result, hydrogen bonds are altered, and water molecules can then bind to the released hydroxyl groups. However, if the temperature is lower than that of the starch gelatinization, the granules are not being damaged but develop new properties, which are dependent on the specific botanical origin of the starch, temperature of annealing, and the concentration of starch in water solution [150,151].

During the starch annealing, their crystallization degree increases (together with the strength of granules structure), which in turn causes the increase in temperature of gelatinization [148,152].

It was further discovered that double helices of potato starch can undergo elongation after annealing, whereas in maize starch, it can induce the formation of new double helices [153,154].

According to the presented data, it can be concluded that the resistance of some types of starch to the enzymatic activity of amylases is determined by the crystalline structure of starch granules, especially the crystallinity of type B.

#### 3.4.5. Resistant Starch Type III

Resistant starch of the third type (RS3) is created during the precipitation of starch paste or gel in the retrogradation process [155]. During the starch gelatinization, partial depolymerization occurs, which results in the formation of a colloidal water solution, often referred to as starch paste. At the specific concentration of amylose and amylopectin (1.5% and 10%, respectively) and at the appropriately low temperature, the gelatinization process of the starch paste occurs. There are two main stages of this process: separation of phases resulting in the formation of the solid polymer phase bound to the liquid phase, and the development of the double helices in the polymer phase. The amylose gel has a structure of microporous threads (diameter of 10–30 nm), which are composed of the joint amylose chains (double helices) with degree of polymerization in the range of 30–70 [156]. Just after a few hours, the highly thermostable (dissolving temperature above 150 °C) β-type crystalline structure is formed due to aggregation of the double helices [155].

Contrary to the amylose, the crystalline structures of amylopectin display lesser stability, which is the primary reason for the lower length of their chains and the dissolving temperature (range from 30–80 °C). Nevertheless, the resistance to the amylolytic enzymes remains only a minor part of the retrograded starch that can undergo enzymatic hydrolysis [157,158,159].

#### 3.4.6. Resistant Starch Type IV

Physical (i.e., thermal treatment) and chemical modifications (or both) of starch result in the formation of resistant starch of the fourth type (RS4). Resistance of these modified starches increases with a higher degree of their substitution with different functional groups [150,160]. Such properties of resistant starch were observed in the starch phosphates, in which the resistance degree increases together with a degree of substitution with phosphoric acid (V) [161].

Another process that occurs during the physical and chemical treatment of starch is dextrinization [162]. It takes place at high temperatures and under the influence of acidic catalysts. Dextrins produced in proper conditions can exhibit properties similar to those of resistant starch. It was observed that the degree of dextrinization and the time of process have a significant influence on the resistance of the acquired dextrin. With increased time of the dextrinization, the number of the 1,3 and 1,2 glycosidic bonds also increases, resulting in a structure more resistant to amylolytic enzymes [162].

#### 3.4.7. Resistant Starch Type 5

Interactions of starch molecules with certain substances (penetration of the amylose helices or formation of complexes) such as lipids or fatty acids may also result in the formation of resistant starch—RS5—in this case. It was observed that complexes of starch with monoglycerides of fatty acids in starch paste exhibit higher resistance to the activity of amylases [162]. Fatty acids are also able to form complexes with amylose chains, which result in the formation of starch resistant to amylolytic hydrolysis. A similar phenomenon is also visible in the small intestine of humans where, due to the activity of lipase, fatty acids are disconnected from lipids and can freely bind with the partially hydrolyzed molecules of starch (or products of starch hydrolysis). As the result, a higher amount of partially or not-digested starch can pass to the colon and be used by the bacteria [163,164].

#### 3.4.8. Resistant Starch in Clinical Trials

Data presented in Table 5 provide evidence that resistant starch is a beneficial addition to daily diet, due to its health-promoting properties. Due to the retrogradation process, the caloric output of the consumed product is lowered since the digestibility of starch was decreased. When the consumption of non-digestible and digestible starch was compared, it was shown that, in the case of non-digestible starch, the levels of glucose and insulin are significantly lower. Furthermore, due to the activity of bacteria present in the colon, resistant starch was utilized (fermentation), which resulted in the production of SCFAs, methane and hydrogen [165]. SCFAs decrease luminal pH and affect the balance of microflora colonizing the large intestine, stimulating the development of beneficial groups of bacteria and reducing the number of pathogenic microorganisms, as described in details in the previous sections of this article.

## 4. Conclusions

In recent years, the surge in lifestyle-originated health problems, such as the pandemic of obesity and other diseases directly or indirectly linked to metabolic disorders, has started to become severely evident. For this reason, it has become particularly important to investigate every possible solution that can limit or prevent such a rapid spread. Seeing an increasing number of studies that associate intestinal microbiota with metabolic disorders, it is obvious that it is not a neglectable factor. Neglected balance of gut microbiome can lead to various diseases, i.e., obesity, type 2 diabetes, irritated bowel syndrome, chronic bacterial prostatitis, and various types of cancers, and can also significantly impair the immune system. In order to achieve a proper balance of intestinal microbiota, a well-thought-out diet is necessary. Probiotics, prebiotics, and certain dietary fibers are proven to be a valid way to support the microbial balance. These bacterial strains and specific compounds possess several health-promoting properties, e.g., anti-obesity or immunological effects. Both resistant starch and dextrins were proven in various independent clinical trials to have positive effects on the subjects such as reduction of BMI, total body fat, and markers of metabolic disorders. Moreover, they increased counts of beneficial gut microorganisms and increased concentrations of short-chain fatty acids and various others highlighted in this review. These outcomes of RS and RD consumption are greatly similar to those of prebiotics. Nevertheless, the number and quality of studies in this field is still not sufficient. The important matter that also must addressed is the influence of gender on the effectiveness of prebiotic and dietary fiber supplementation, which is omitted in the vast majority of studies. Such knowledge may potentially be of great importance in the field of improving the efficacies of prebiotic treatments. Moreover, during research, the number of tested subjects is usually relatively low, mostly due to legal requirements and lack of volunteers. When combined with the restricted ability of supervision of the participants and the long time required for the studies, a high probability of errors is created. There is a real potential in the usage of RS and RD supplements, which are easily produced and accessible to the public. Accordingly, additional research is required to fill the void of knowledge on RS and RD and their association with human health to fully utilize their capabilities.

## Figures and Tables

**Table 1:** Summary of various standard methods used for dietary fiber determination.

AOAC Method	Measurement Type	Enzymes Used	Other Properties
985.29	Total HMWDF	Bacterial α-amylase, protease, and amyloglucosidase	1 g sample, phosphate buffer
991.42	IDF in food products	Bacterial α-amylase, protease, and amyloglucosidase	1 g sample, phosphate buffer
991.43	IDF and SDFP	Bacterial α-amylase, protease, and amyloglucosidase	1 g sample, Tris or MES buffer, pH = 8.2
993.19	SDFP in food products	Bacterial α-amylase, protease, and amyloglucosidase	1 g sample, phosphate buffer
993.21	HMWDF (samples with more than 10% fiber and less than 2% starch)	Carried out without enzymes	0.1 g sample, without buffer,
994.13	HMWDF	Bacterial α-amylase and amyloglucosidase	0.25–0.5 g sample, acetate buffer, also provides composition of sugars and Klason lignin
2001.03	HMWDF and SDFS	Bacterial α-amylase, protease, and amyloglucosidase	1 g sample, phosphate buffer, only for samples free of resistant starch
2009.01	HMWDF and SDFS	Pancreatic α-amylase, protease, and amyloglucosidase	1 g sample, meleate buffer, pH = 6.0, available for any kind of food
2011.25	IDF, SDFP, and SDFS	Pancreatic α-amylase, protease, and amyloglucosidase	1 g sample, meleate buffer, pH = 6.0, available for any kind of food

HMWDF = higher-molecular-weight dietary fiber; IDF = insoluble dietary fiber; SDFP = dietary fiber that dissolves in water but not in 76% aqueous ethanol; SDFS = dietary fiber that is soluble in both water and in 76% aqueous ethanol. Total dietary fiber = HMWDF + SDFS; HMWDF = IDF + SDFP.

**Table 2:** Supplements containing dietary fibers available on the market.

Dietary Fibers	Supplements Available on the Market	Dosage of Fiber Per Serving
Wheat dextrin	Benefiber (Novartis Consumer Health, USA)	3 g
Wheat dextrin	Optifiber (Kirkland Signature, USA)	3 g
Psyllium	3-in-1 FIBER (Metamucil, AUS)	2.4 g
Resistant tapioca dextrin	Completely Clear Organic Prebiotic Fiber (RenewLife, USA)	6 g
Chia seed, organic flaxseed	Flax & Chia (Whole Foods Market, USA)	8 g
Dextrin	Fiber Powder (Equate, KW)	3.5 g
Flax seed, chia seed, sunflower seed and others	Raw Organic Fiber (Garden of Life, USA)	9 g
Soluble stabilized rice bran, rice germ, chicory root	Zeal for Life (Zurvita, USA)	4 g
Polydextrose	Fiber Well Fit (Vitafusion, USA)	4 g
Chicory, inulin	Daily Fiber Gummies (Konsyl Pharmaceuticals, USA)	3 g
Flax seed, chia seed	Super Seed Beyond Fiber (Garden of Life, USA)	6 g
Chicory root, tapioca starch	Prebiotic Fiber Gummies (Lifeable, USA)	4 g
Psyllium	Psyllium Husk (Sunergetic, USA)	1.5 g
Soluble corn fiber	Prebiotic Soluble Fiber (Just Better, USA)	5 g

**Table 3:** Clinical trials on the effect of resistant dextrins on various health markers.

Reference	Dextrin	Patients	Dosage [g/Day]	Time of Study	Outcome
[125]	Nutriose^®^	55 women with type-2 diabetes (age 30–65)	10 g	8 weeks	Significant decrease in fasting insulin, malondialdehyde (MDA), and endotoxin.
[126]	Nutriose^®^	62 women with polycystic ovary syndrome (age 18–45)	20 g	3 months	Positive influence on metabolic parameters, androgen levels, hirsutism, and menstrual cycle regularity
[127]	Nutriose^®^	62 females (age 18–45)	20 g	12 weeks	Confirmed the positive and significant effects in reducing anthropometric indices
[128]	Nutriose^®^	65 females with type-2 diabetes (age 30–65)	10 g	8 weeks	Improved end products of advanced glycation and other risk factors of cardiometabolic diseases
[129]	Nutriose^®^	55 females with type-2 diabetes (age 30–65)	10 g	8 weeks	Supplementation yielded significant decrease in levels of cortisol, LPS. Increased levels of CD8 lymphocytes. Improved mental health and immune response.
[130]	Nutriose^®^	50 males, 50 females (age 35–55)	8 g, 14 g, 18 g, 24 g	3 weeks	Decreased hunger over longer supplementation. Significant increase of short-term satiety
[131]	Nutriose^®^	50 males, 50 females (age 35–55)	8 g, 14 g, 18 g, 24 g	9 weeks	Significant reduction of energy intake, BMI, and body fat in groups with intake of 14–24 g Nutriose per day
[132]	Resistant maltodextrin and isomaltose	27 males, 14 females (age 18–80)	5.28 g and 16.5 g	20 weeks	Improvement of insulin resistance in patients with type-2 diabetes, overweight, and obesity
[36]	Resistant dextrin (MPCIR)	38 males, 61 females (age 45–70)	8–34 g	12 weeks	Improvement of glycemic control, insulin resistance, and blood pressure.
[133]	Resistant maltodextrin and isomaltose	11 males, 3 females (age 18–80)	5.28 g and 16.5 g	20 weeks	Enhancement of pathways related to metabolism, including terpenoid-quinone, lipopolysaccharides, and *N*-glycan biosynthesis. Significant impact on gut microbiota in diabetic subjects.
[134]	Resistant dextrin	275 subjects (meta-analysis)	10–34 g	8–12 weeks	Beneficial effects on BMI and weight loss in overweight adults.
[49]	Nutriose^®^	22 males, 64 females (age 18–59)	10–20 g	2 weeks	Increased counts of *Bacteroides* spp. and inhibition of *Clostridium perfringens.* Increased β-glucosidase activity and decreased colonic pH. No indications of gastrointestinal intolerance were found.
[48]	Nutriose^®^	17 males, 19 females (age 22–55)	14 g	4 weeks	Supplementation was associated with higher fasted satiety scores and attenuation of the glycemic response
[135]	Isomaltulose, resistant dextrin, and inulin	8 males, 22 females (age 18–60)	45 g in total	4 days	Reduction of glycemic response and longer term of satiety without causing any serious side effects
[136]	Fructooligosaccharides, xylooligosaccharides, polydextrose, and resistant dextrin	90 males, 50 females (age 40–75)	30 g total (7.5 g each component)	1 week	Improved serum immunologic indicators
[137]	Nutriose^®^	120 overweight males (age 26–35)	34 g	12 weeks	Reduction of energy intake, BMI, body fat percentage, and waist circumference. Improved glucose metabolism markers. Improved lipid metabolism. No adverse effects.
[138]	Nutriose^®^	12 males (age 20–65)	50 g	10 h	Decreased ghrelin concentrations in response to the lunch, prolonged energy release. Reduced glycemic and insulinemic responses to breakfast.
[34]	Fibersol-2	23 males, 28 females (age 19–33)	25 g	3 weeks	Increased fecal *Bifidobacteria* counts and stool wet weight
[139]	Fibersol-2	10 males, 9 females (age 20–65)	5 or 10 g	4 h	A total of 10 g of Fibersol-2 stimulates production of satiety hormones (peptide-YY).
[140]	Fibersol-2	HTC116 cell line	-	-	Significant inhibition of tumor growth of HCT116 cells by induction of apoptosis without visible signs of toxicity in vivo.
[141]	Fibersol-2	19 subjects	5 or 10 g	1 day	FS-2 administration stimulated production of specific satiety peptides such as PYY, decreased the hunger peptide ghrelin, and enhanced satiety after a meal.
[33]	Fibersol-2	32 males, 34 females (age 18–30)	15 g	3 weeks	Improved colonic functions, transit time, stool volume, and consistency.
[142]	Fibersol-2	4 males, 28 females (age 32–63)	20 g	20 days	Improved symptoms of the idiopathic primary chronic constipation.
[143]	Fibersol-2	20 males, 10 females (age 50–72)	27 g	12 weeks	Improved state of the risk factors of metabolic syndrome through the reduction of visceral fat and improvement of glucose and lipid metabolism.
[144]	Fibersol-2	24 males (age 20–24)	11 g	4 days	Increased satiety

Product information: Nutriose® Soluble Fiber, Roquette, France; Fibersol®, ADM/MATSUTANI LLC, USA.

**Table 4:** Short summary of resistant starch types and their sources.

Type of Resistant Starch	Description	Production	Source
RS1	Protected physically, trapped in matrix resistant to enzymatic digestion	Milling or partial grinding	Grains, seeds, legumes, pastas
RS2	Raw starch granules with crystalline structure of type B.	Gelatinization by thermal treatment	Green bananas, raw potatoes, corn with high amylose content, specific legumes
RS3	Starch that underwent retrogradation process	Retrogradation by repeated thermal treatment (cooking and cooling)	Starch products that underwent specific thermal treatment (breads, cakes, cornflakes)
RS4	Chemically or physically modified starches, cross-linked with chemical reagents	Mainly chemical, enzymatic, or thermal treatment often resulting in substitution with phosphates	Hardly available for human consumption. Present in specially designed starch products and food additives.
RS5	Amylose complexes with lipids or fatty acids	Formed during food processing or naturally occurring within foods of high amylose content	Products with high amylose content

**Table 5:** Clinical trials on the effect of resistant starch on various health markers.

Reference	Resistant Starch	Patients	Dosage [g/Day]	Time and Type of Study	Outcome
[166]	MSPrebiotic^®^	24 females, 18 males (age ≥ 70) and 25 females, 17 males (age 30–50)	30 g/day	3 months/RCT study	Significant reduction of insulin resistance, which is an important risk factor for developing type-2 diabetes.
[167]	RS2: Hi-maize 260, National Starch LLC	56 females with type 2 diabetes (age 32–65)	10 g/day	8 weeks/RCT study	Significantly decreased levels of MDA, glycosylated hemoglobin, insulin, improved homeostasis model of insulin resistance and lowered endotoxins levels, a significant increase in TAC and glutathione peroxidase
[168]	Cross-linked RS type 4	7 females, 6 males (age 22–32, BMI 22–28)	27 g/day	1 day/ RCT study	Peak glucose and insulin concentrations in subjects were decreased
[169]	VERSAFIBE™ 2470	14 males, 14 females (age 24–58)	11.6 g/day	1 day/ RCT study	Significant reduction in postprandial serum glucose and decrease in maximum glucose concentration. Reduced postprandial serum insulin.
[170]	High-amylose maize type 2 resistant starch	11 males, 22 females (age 18–69, BMI < 35)	15, 30 g/day	4 weeks/ RCT study	Improved insulin sensitivity in male subjects.
[171]	High-amylose maize (RS2)	20 males, 39 females (BMI ≥ 27, age 35–75)	45 g/day	12 weeks/ RCT study	Reduced the inflammatory marker TNF-α and heart rate, but no significant improvement of glycemic control and other cardiovascular disease risk factors
[172]	RS4-enriched flour (30% *v*/*v*)	86 adults (gender not specified)	25.7 g/day	12 weeks/RCT study	No significant effect for glycemic variables and blood pressures. Improved dyslipidemia (lowered cholesterol levels) and body composition.
[173] (Meta-analysis)	Resistant starch	13 studies, 15–75 subjects per study	-	4–14 weeks	Improved inflammatory biomarkers
[35] (Meta-analysis)	Resistant starch	19 studies, 1014 subjects in total	-	-	Significant reduction in fasting plasma glucose, insulin, total cholesterol, and tumor necrosis factor alpha.
[174]	HAM-(RS2)	16 males, 8 females (BMI = 30, average age 55)	25 g	57 days	Improved glycemic efficiency and fasting insulin sensitivity in adults at increased risk of T2D
[175]	Resistant starch	19 males, 31 females (age > 50, overweight)	25 g	12 months	Glycemic control in prediabetic patients was unaffected by RS-rich diet in contrast to the regular fibre rich diet.
[176]	Resistant starch in form of cocoa and unripe banana flour beverage	60 females (age 20–50)	30 g	6 weeks	Decreased the symptoms of dyspepsia, improved gastrointestinal symptoms, and increased production of propionic acid. The cocoa beverage showed an anti-inflammatory effect.
[177]	Arabinoxylan and resistant starch	14 males, 5 females (age 39–75)	21 g	4 weeks	Improved fasting LDL and total cholesterol. No diet related impact on postprandial lipaemia.
[36]	Milk powder co-supplemented with inulin and resistant dextrin	38 males, 61 females (age 45–70)	45 g	12 weeks	Supplementation improved glycemic control, insulin resistance, and blood pressure.
[178]	Resistant starch Hi-Maize^®^	18 males, 13 females (age 42–65)	16 g	4 weeks	Supplementation improved inflammation and oxidative stress and reduced indoxyl sulfate plasma levels
[179]	HAM-resistant starch type 2	28 males, 16 females (age 41–74)	25 g	8 weeks	Significant reduction of levels of inflammatory and oxidative markers in hemodialysis patients
[180]	HAM-resistant starch type 2	29 males, 21 females (age 43–71)	25 g	8 weeks	Decreased serum levels of serum creatinine and p-cresol
[181]	Resistant starch (potato starch and high-amylase starch)	39 males, 31 females (age 18–80)	50 g	12 weeks	Improvement of the blood glucose and blood lipid levels, decrease in the serum uric acid (UA) and urine β2-MG, and reduced antioxidative stress
[182]	Green banana biomass	26 males, 87 females (age 18–85)	40 g (approx. 5 g of resistant starch)	24 weeks	Consumption of bioactive starches can improve metabolic control and body composition
